# Influenza

**Published:** 1848-04

**Authors:** 


*Influenza.*—This epidemic has been very extensively prevalent in Great Britain and throughout Europe. At Lille, 10,000 persons are said to be affected by it: at Toulouse, upwards of 15,000: whilst at Marseilles, out of a population of 160,000, at least half are suffering from it.—*Annalist.*

